# Integrated analysis of microbiome and metabolome reveals insights into cervical neoplasia aggravation in a Chinese cohort

**DOI:** 10.3389/fcimb.2025.1556153

**Published:** 2025-05-08

**Authors:** Qingzhi Zhai, Luyang Zhao, Mingyang Wang, Li Li, Li-an Li, Mingxia Ye, Mingxia Li, Chengfeng Xu, Yuanguang Meng

**Affiliations:** ^1^ Department of Obstetrics and Gynecology, The Seven Medical Center of Chinese People's Liberation Army General Hospital, Beijing, China; ^2^ Emergency Department, The Fourth Medical Center of Chinese People's Liberation Army General Hospital, Beijing, China

**Keywords:** cervical cancer, cervical squamous intraepithelial lesion, metabolome, microbiome, biomarkers, integrated analysis

## Abstract

**Introduction:**

Cervical carcinoma (CC) remains one of the significant cancers threatening women's health globally. Increasing evidence suggests that alterations in the microbiota are closely associated with cancer development. However, the understanding of reliable biomarkers and underlying mechanisms during the aggravation of cervical neoplasia such as cervical intraepithelial neoplasia (CIN) and CC is still relatively limited.

**Methods:**

In this study, cervical swab samples from 53 healthy controls, 51 high-grade squamous intraepithelial lesion (HSIL), and 52 CC patients were subjected to 16S rDNA sequencing and metabolomics analysis.

**Results:**

We observed significant differences in the cervical microbiota between CC patients and healthy controls or HSIL groups. Compared to the healthy controls, CC patients exhibited increased microbial diversity, decreased abundance of *Lactobacillus*, and notable changes in microbial composition. Metabolomics analysis revealed significantly elevated levels of the inflammatory mediator Prostaglandin E2 (PGE2) in CC samples. Through random forest modeling and ROC curve analysis, we identified a combination of key microbiota (*Porphyromonas*, *Pseudofulvibacter*) and metabolites (Cellopentaose, PGE2) as diagnostic biomarkers with high diagnostic value for CC. Furthermore, we found a significant correlation between the cervical microbiota *Porphyromonas* and the metabolite PGE2, suggesting a potential role of key microbiota in inducing inflammation.

**Discussion:**

These findings indicate that alterations in cervical microbiota and metabolites may be closely associated with the occurrence and aggravation of cervical neoplasia, providing new insights for further understanding the mechanisms of cervical neoplasia progression and developing novel diagnostic markers and therapeutic approaches.

## 1 Introduction

Cervical carcinoma (CC) stands as the fourth most common cancer among women globally (Sung et al., 2021), underscoring the critical importance of early detection and treatment for improving patient outcomes. Despite notable strides in pinpointing diagnostic, prognostic, and predictive biomarkers for CC, there remains an incomplete understanding of reliable markers and their underlying mechanisms (Volkova et al., 2021). In recent years, with advancements in sequencing technologies and LC-MS metabolomics, interest has surged in investigating microbiota and metabolites as biomarkers for human cancer.

Research on microbiota-related biomarkers suggests that the composition of cervical microbiota is significantly altered in CC patients compared to healthy individuals (Mulato-Briones et al., 2024; Li et al., 2024). Recent studies have reported cervical and vaginal microbiota changes with precancerous cervical lesions and cervical dysplasia (Natalia et al., 2024; Johanna et al., 2024; Zhang et al., 2024). While the vaginal microbiota of healthy individuals is dominated by *Lactobacillus*, CC groups exhibit an overrepresentation of *Firmicutes* and *Bacteroidetes* (Mulato-Briones et al., 2024). Similarly, studies have shown a decrease in *Lactobacillus* abundance in patients with cervical lesions and CC (Kovachev, 2020; Norenhag et al., 2020). Sequencing of vaginal microbiota in different stages of cervical lesions revealed a gradual decrease in *Lactobacillus* abundance with lesion progression (Wei et al., 2022). *Lactobacillus* may play a crucial role in maintaining vaginal microbial balance, potentially exerting anti-HPV and anti-inflammatory effects (Xu et al., 2022). Moreover, CC patients exhibit significantly increased diversity in cervical microbiota compared to healthy controls (Zeber-Lubecka et al., 2022). Microbial-induced inflammation is believed to be a key driver in CC development (Zhou et al., 2021).

Metabolomics offers promising screening markers for the detection, screening, early diagnosis, and treatment of CC. During tumorigenesis and progression, tumor cells undergo significant metabolic reprogramming (Morandi et al., 2017). Metabolic alterations in patients with cervical lesions mainly involve glucose, amino acid, lipid, nucleotide, purine, and choline metabolism (Jia et al., 2023). Microbiota may influence host cervical cell energy metabolism and metabolic pathways, potentially contributing to cancer development; however, the specific microbial contributions remain largely unknown.

As exploration into microbiota and metabolomics as potential biomarkers for cervical cancer advances, our comprehension of diagnostic approaches for CC becomes more profound. A study involving a cohort of 10 low-grade squamous intraepithelial lesion (LSIL), 10 high-grade squamous intraepithelial lesion (HSIL), 10 CC, and 10 healthy controls detected significant alterations in *Lactobacillus*, *Prevotella*, and *Aquabacterium*, alongside shifts in lipids and organic acids during lesion development. However, the relatively small sample size in this cohort constrains the reliability of biomarker identification and mechanistic investigations (Xu et al., 2022). The understanding of the interplay between gut microbiota, metabolites, and their roles in CC’s onset and progression remains limited. In this study, we conducted microbiota and metabolomics analysis on cervical swab samples from 51 Normal, 48 HSIL, and 52 CC cases to uncover changes in cervical microbiota and metabolites and their interconnections. Furthermore, we delved into potential molecular pathways to enhance understanding of cervical lesion occurrence and aggravation.

## 2 Materials and methods

### 2.1 Sample collection and preparation

The samples for this study were collected from the Department of Obstetrics and Gynecology at the First Medical Center of the Chinese People’s Liberation Army General Hospital. Participants were recruited openly from female patients who sought medical attention at our department from January to June in the year 2023, within the age range of 30 to 73 years. Inclusion criteria: (1) Patients diagnosed via cervical cytology, HPV screening, and colposcopy, or healthy controls with negative cervical findings; (2) No sexual activity or intravaginal medication within 3 days prior to sampling; (3) No oral or intravenous anti-inflammatory therapy within 1 week prior to sampling. Exclusion criteria: (1) Advanced-stage CC patients; (2) Vaginal disinfection or other treatments prior to sampling; (3) Pregnancy or lactation; (4) Vulvovaginal candidiasis (VVC), bacterial vaginosis (BV), trichomoniasis (TV) (Qingzhi et al., 2022), or aerobic vaginitis (AV) diagnosed by GY66 automatic vaginal microecological analyzer (Qingzhi et al., 2022); (5) Oral hormonal therapy. A total of 156 cases were included for subsequent analyses. A total of 156 cases were ultimately included for 16S rRNA gene amplicon sequencing and untargeted metabolomic analysis using ultra-high-performance liquid chromatography coupled with high-resolution mass spectrometry (UHPLC-HRMS). The cohort comprised 52 cases of CC, 51 cases of HSIL, and 53 healthy controls (Normal). All CC and HSIL cases were confirmed through pathological diagnosis. Cervical swabs were directly placed into cryovials, stored in liquid nitrogen, and utilized for both amplicon sequencing and non-targeted metabolomic profiling.

### 2.2 Cervical sampling procedure

Excessive cervical secretions were wiped, and a disposable sampling swab was inserted into the cervical canal and gently rotated clockwise for 3–5 turns. Subsequently, the cervical swab was withdrawn and placed into a cryotube; excess swab tail was broken off at the tube opening, leaving the swab head in the sampling tube, which was then stored in liquid nitrogen. Dry ice was used for transportation to the experimental personnel at Novogene (Novogene, Beijing, China), for untargeted metabolomic analysis and 16S sequencing.

### 2.3 Metabolomics profiling

A total of 400 μL extraction solution (methanol: acetonitrile: water = 2:2:1, v/v, containing isotope-labeled internal standard mixture) was added to each sample. Subsequently, the samples were immersed in liquid nitrogen for 1 minute, followed by thawing and vortexing for 30 seconds. This step was repeated three times and then ultrasonication for 10 minutes in an ice-cold water bath was performed. After incubating at -40°C for 1 hour, the samples were centrifuged at 4°C, 12,000 rpm for 15 minutes. The supernatant was collected for subsequent analysis. An amount of 5 μL supernatant from all samples were mixed to create a quality control sample (QC), and a total of 10 QC samples were prepared. During the analysis of the samples, a QC sample was inserted every 10 samples for quality control monitoring.

Metabolites extracted were chromatographically separated using an ACQUITY UPLC BEH Amide column (2.1 mm × 100 mm, 1.7 μm; Waters, Milford, MA, USA) on a Vanquish ultra-high-performance liquid chromatography system (Thermo Fisher Scientific). The mobile phase A consisted of aqueous solution containing 25 mmol/L ammonium acetate and 25 mmol/L ammonia solution, while mobile phase B was acetonitrile. The sample tray was maintained at 4°C, and the injection volume was 2 μL. Mass spectrometry was performed using a Thermo Q Exactive HFX mass spectrometer, and data acquisition was controlled by the Xcalibur™ software (3.1.66.10; Thermo Fisher Scientific, Inc.). Detailed parameters were as follows: Sheath gas flow rate: 30 Arb, Aux gas flow rate: 25 Arb, Capillary temperature: 350°C, Full MS resolution: 60,000, MS/MS resolution: 7,500, Collision energy: 10/30/60 in NCE mode, Spray Voltage: 3.6 kV (positive) or -3.2 kV (negative).

The raw data obtained were pre-processed using the CD data processing software (Thermo Fisher Scientific, Inc.). Compound exact masses were determined from the high-resolution extracted ion chromatogram (XIC) using the mass-to-charge ratio. Predicted molecular formulas were generated based on mass number deviations and adduct ion information. Compound identification was performed by matching with fragment ions, collision energy, and other information for each compound in the high-quality mzCloud database (https://www.mzcloud.org/home), complemented by information from the mzVault and MassList databases. Compounds with a coefficient of variance (CV) in QC samples of less than 30% were selected for final identification. The peak area of each feature represented the relative quantification of a compound, and the total peak area was used for normalization. The final quantification results for the metabolites were obtained. Functional and classification annotation of identified metabolites was conducted using major databases, including KEGG (Kanehisa and Goto, 2000) and HMDB (https://hmdb.ca/metabolites). Orthogonal Partial Least Squares Discrimination Analysis -Discriminant Analysis (OPLS-DA) was then performed (Thevenot et al., 2015), and the differential metabolites between two groups were estimated using variable importance in projection (VIP). The threshold for the identification of differential metabolites was set as VIP > 1 and *P-value* < 0.05 based on a Student’s t-test.

### 2.4 DNA extraction and 16s rDNA sequencing

Genomic DNA was extracted from the collected samples using the CTAB method. The concentration of DNA was quantified using a Nanodrop spectrophotometer, and its purity and integrity were assessed via 1% agarose gel electrophoresis. Subsequently, the extracted DNA was diluted to a final concentration of 1 ng/μl using sterile water. Amplification of the 16S rRNA genes was conducted with 341F/806R primers that incorporated barcodes for sample identification (Guo et al., 2017). PCR reactions were performed in 30 μL volumes, comprising 15 μL of High-Fidelity PCR Master Mix (New England Biolabs), 0.2 μM of both forward and reverse primers, and approximately 10 ng of template DNA. The resulting amplicons were combined in equal proportions and purified using the TIANgel Purification Kit (TIANGEN Biotech). Sequencing libraries were then constructed utilizing the TIANSeq Fast DNA Library Prep Kit (Illumina) provided by TIANGEN Biotech. Library quality was assessed using the Qubit@ 2.0 Fluorometer (Thermo Scientific) and the Agilent Bioanalyzer 2100 system. Finally, the prepared library was sequenced on the Illumina platform, following the 2 × 250 bp paired-end sequencing protocol. All primers were designed by multiPrime at http://www.multiprime.cn (Xia et al., 2023).

### 2.5 Microbiome analysis

Microbiome bioinformatics were performed with QIIME 2 (Caporaso et al., 2010) with slight modification according to the official tutorials. Briefly, raw sequence data were demultiplexed using the demux plugin following by primers removed with cutadapt software (Martin, 2011). Sequences were then quality filtered, denoised, merged and chimera removed using the DADA2 plugin (Callahan et al., 2016). Species annotation was performed using QIIME2 software with Silva Database (Pruesse et al., 2007). Cervicovaginal microbiome community state types (CSTs) were assigned using the VALENCIA tool (Michael et al., 2020) to assign CSTs.

Venn diagram of amplicon sequence variants (ASVs) from different groups was drawn using VennDiagram package (Chen and Boutros, 2011). Alpha diversity, including Chao1 and Shannon indices were calculated with QIIME2 software (Caporaso et al., 2010). Principal Coordinate Analysis (PCoA) on weighted unifrac distances among samples was performed (Lozupone and Knight, 2005) and visualized using ade package (Bougeard and Dray, 2018) and ggplot2 package (Wickham et al., 2008) in R software (Version 3.6.2). The indicator values (IndVal) of all taxa in each group were calculated using labdsv package (Roberts, 2016) to determine the indicator species in each group.

### 2.6 Integration of multi-omics data

Spearman correlation coefficient between key indicator genera and metabolites was calculated using psych package (Revelle, 2015), and interaction relationships with absolute correlation value greater than 0.3 and *P-value* less than 0.05 were filtered, which were then visualized using Cytoscape (Shannon et al., 2003).

### 2.7 Statistical analysis

The clinical factors including age and BMI were compared among groups using one-way analysis of variance (ANOVA) test. Inter-group differences between two groups in the alpha diversity indices were analyzed with Kruskal-Wallis test, and a Benjamini-Hochberg corrected *P-value* < 0.05 indicated significant difference. To identify differences of microbial communities between two groups, STAMP software was utilized with default parameters.

## 3 Results

### 3.1 Subjects’ information

The samples in this study were all collected from the Department of Obstetrics and Gynecology, First Medical Center of the Chinese People’s Liberation Army General Hospital. The female participants’ ages ranged from 30 to 73 years old. Specifically, there were 53 cases in the normal group, 51 cases in the HSIL group, and 52 cases in the CC group. No significant differences in age and BMI were observed among the three sample groups (**Table 1**). Notably, the CC group exhibited an older age profile (median: 49 years) compared to the HSIL group (median: 46 years), consistent with the epidemiological trend of cervical cancer progression (**Supplementary Figure 1**). The majority of patients in the CC and HSIL groups were infected with one or more high-risk HPV types (**Table 1**; **Supplementary Table 1**). In the cohort of CC specimens, the predominant histologic subtype was squamous cell carcinoma (39 cases), with a smaller portion being adenocarcinomas (13 cases) (**Table 1**; **Supplementary Table 1**). The tumor stages were predominantly classified as Stage I (30 cases), followed by Stage II (11 cases), and Stage III (8 cases), with only 3 cases in Stage IV (**Table 1**; **Supplementary Table 1**). For the HSIL samples, the staging primarily comprised CIN2 (20 cases), CIN2-3 (18 cases), and CIN3 (13 cases) (**Table 1**; **Supplementary Table 1**). The chi-square test was further employed to compare the proportions of individuals with different menopausal status among groups, which indicated that there were no significant differences (*P-value* > 0.05) in the pairwise comparisons between the Normal, HSIL, and CC groups.

**Table 1:** Statistical summary of patient age, BMI and HPV infection.

	n	Normal (n=53)	HSIL (n=51)	CC (n=52)	*P-value*
BMI (mean(SD))	156	23.05 (2.83)	22.28 (3.42)	22.32 (1.64)	*0.27*
Age (mean(SD))		48.66 (6.35)	46.33 (7.34)	49.35 (7.15)	*0.074*
HPV status					
HPV 16 positive		0 (0)	22 (41.51%)	34 (65.38%)	*0.004*
HPV 18 positive		0 (0)	2 (3.77%)	13 (25.00%)	*0.006*
other high-risk		0 (0)	18 (33.96%)	6 (11.54%)	*0.009*
Single high-risk		0 (0)	37 (69.81%)	46 (88.46%)	*0.073*
Multiple high-risk		0 (0)	2 (3.77%)	4 (7.69%)	*0.692*
Histotype				Squamous Cell Carcinoma (39) Adenocarcinoma (13)	
Stage			CIN2 (20) CIN2-3 (18) CIN3 (13)	I (30) II (11) III (8) IV (3)	
Menopausal status		Luteal (16) Menopause (23) Follicular (14)	Luteal (16) Menopause (16) Follicular (19)	Luteal (13) Menopause (28) Follicular (11)	*0.207*

Values are presented as mean, with standard deviation (SD) in parentheses for BMI and age. HPV status is shown as number of patients, with percentages (%) in parentheses. Histotype, stage, and menopausal status are presented as number of patients in each category in parentheses. *P-value*s were calculated using Fisher’s exact test for HPV status (HSIL vs. CC) and Chi-square test for Menopausal status across three groups.

An average of 124,569 reads per sample were obtained by 16s amplicon sequencing, with an average quality score exceeding 30 (**Supplementary Table 2**). The mean proportion of effective reads stood at 75.4%, resulting in an average of 119,296 reads per sample post-denoising (**Supplementary Table 3**). In total, 32,317 Amplicon Sequence Variants (ASVs) were identified, averaging 342 ASVs per sample (**Supplementary Table 2**). Untargeted metabolomic profiling was performed, and a total of 2,879 metabolites were detected. Among them, 1,543 metabolites were annotated in the HMDB or KEGG databases.

### 3.2 Diversity of bacterial community

**Figure 1:**
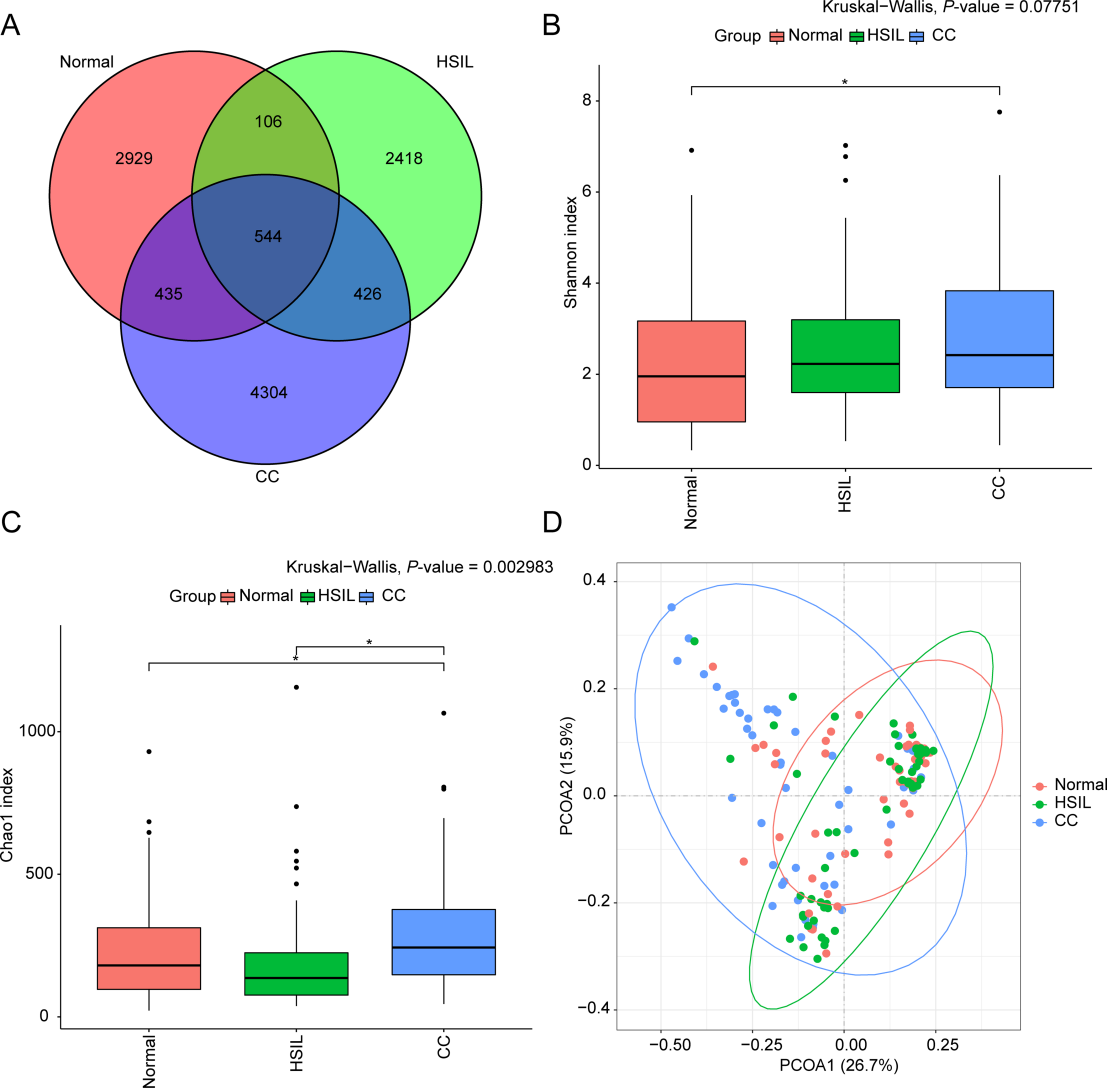
Overview of bacterial community data. **(A)** Venn diagram illustrating the overlap of ASVs in samples from the Normal, HSIL, and CC groups. **(B, C)** Boxplots of Shannon **(B)** and Chao1 **(C)** indices for each group, with inter-group differences assessed by Kruskal-Wallis test. * represents significant differences indicated with Benjamini-Hochberg corrected *P-value* < 0.05. **(D)** Scatter plot of PCoA analysis for samples in each group.

The intersection analysis revealed a relatively modest number of shared ASVs (544 common ASVs) among the three groups: Normal, HSIL, and CC (**Figure 1A**). Inter-group differences were observed in the alpha-diversity indices (Kruskal-Wallis test, Benjamini-Hochberg corrected *P-value* < 0.05) (**Figures 1B, C**). Specifically, the Shannon index in the CC group was significantly higher than that in the Normal group (**Figure 1B**), while the Chao1 index was significantly higher in the CC group compared to both the HSIL and Normal groups (**Figure 1C**). Beta-diversity analysis, based on weighted UniFrac PCoA, indicated that samples from each group clustered together, suggesting differences in microbial structure among these groups (**Figure 1D**).

### 3.3 Bacterial community characterization

**Figure 2:**
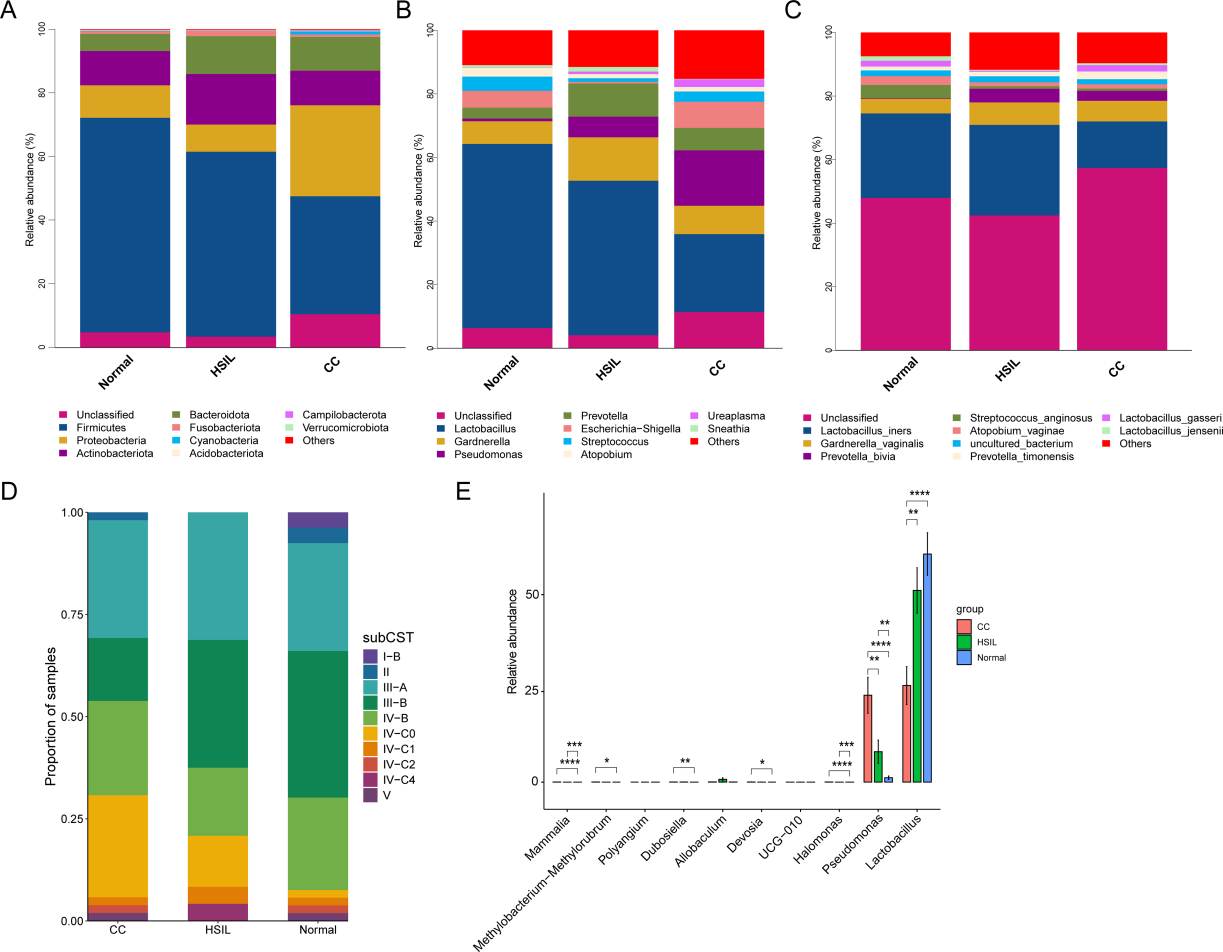
Composition of the microbial community. **(A-C)** Stacked bar charts depicting the taxonomy composition at the phylum **(A)**, genus **(B)**, and species **(C)** levels for different groups. **(D)** Stacked bar plot representing the proportions of samples assigned to each sub-CSTs across all groups. **(E)** Differential analysis at the genus level across the CC, HSIL, and Normal groups using the wilcox method, where FDR-corrected *P-value* < 0.05 indicates significant inter-group differences. *, **, ***, and **** represent *P-value* < 0.05, *P-value* < 0.01, *P-value* < 0.001, and *P-value* < 0.0001, respectively.

The predominant microbiota at the Phylum level in samples from various groups comprised *Firmicutes*, *Proteobacteria*, *Actinobacteriota*, and *Bacteroidota*, etc. Notably, *Firmicutes* exhibited a gradual decrease in abundance as the disease progressed from Normal to HSIL and further to CC, while *Proteobacteria* demonstrated a relatively higher average abundance in the CC group (**Figure 2A**). At the Genus level, the predominant microbiota included *Lactobacillus*, *Gardnerella*, *Pseudomonas*, *Prevotella*, *Escherichia-Shigella*, and *Streptococcus*, and others (**Figure 2B**). Similarly, the average abundance of the *Lactobacillus* also showed a gradual decrease as CC progressed (**Figure 2B**). In contrast, *Pseudomonas* displayed a gradual increase in abundance (**Figure 2B**). Additionally, species with higher abundances were identified, including *Lactobacillus iners*, *Gardnerella vaginalis*, *Prevotella bivia*, *Streptococcus anginosus*, and *Atopobium vaginae* (**Figure 2C**). Among them, the abundance of *L. iners* was lower in CC, compared with the other groups (**Figure 2C**).

The clustering method based on VALENCIA was utilized to analyze the CSTs, resulting in the assignment of ten sub-CSTs. Among all samples, the most prevalent communities were III-A (almost completely L. iners), III-B (less L. iners but still the majority), IV-B (which contains a high to moderate relative abundance of *G. vaginalis* and *A. vaginae*), and IV-C0 (contains low relative abundances of *G. vaginalis*, BVAB1, and *Lactobacillus* spp. and includes a relatively even community with Prevotella spp.) (**Figure 2D**). Inter-group differential analysis indicated that *Lactobacillus* and *Pseudomonas* were significantly lower and higher in the CC group compared to the Normal group, respectively, while no significant different genera were detected among other comparisons (**Figure 2E**).

**Figure 3:**
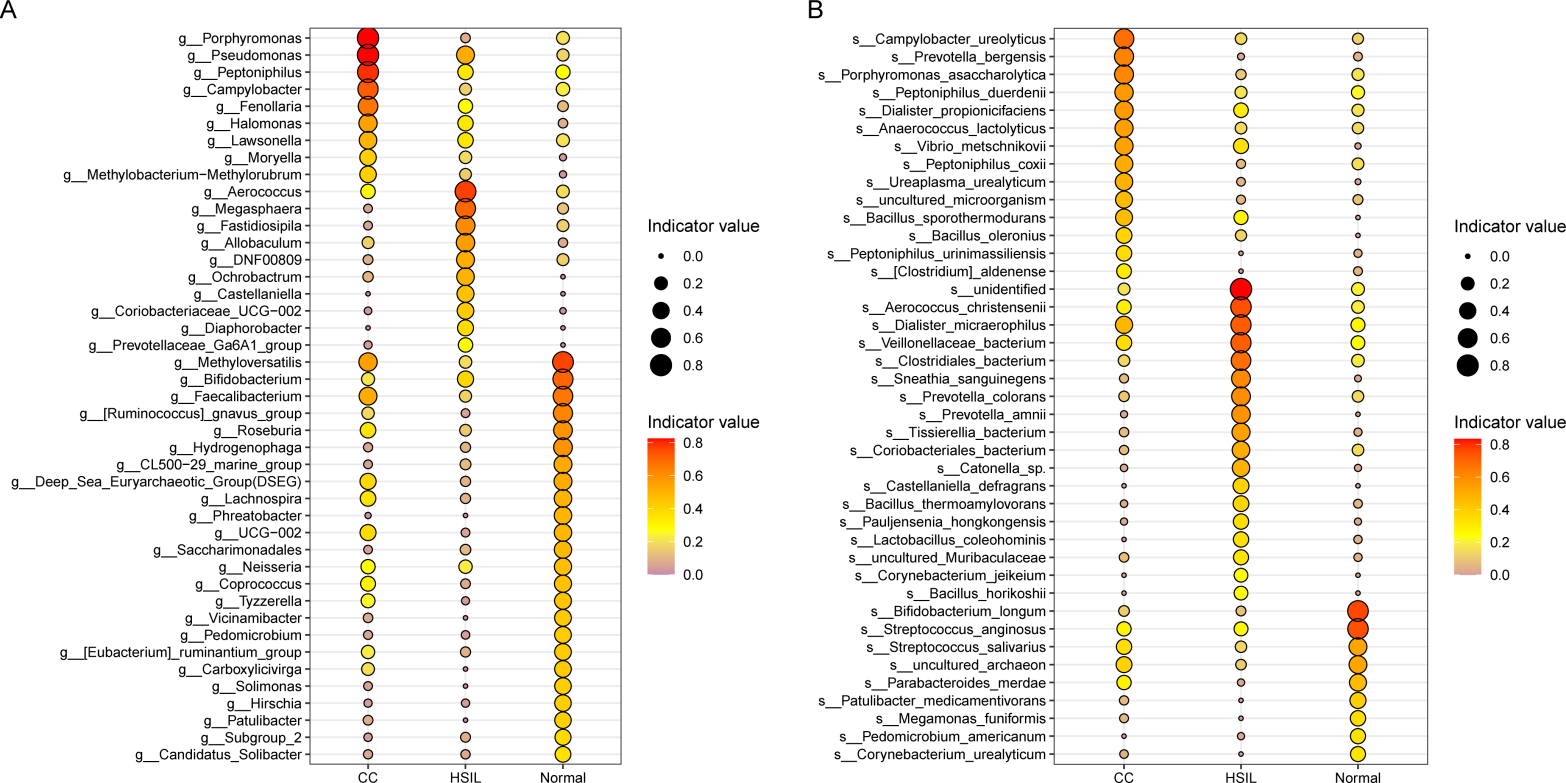
Indicator species analysis. **(A, B)** Species indicator values at the genus **(A)** and species **(B)** levels for different groups, where the size of the points is proportional to the indicator values, and the red color indicates higher indicator values.

To discern microbial biomarkers linked with particular diseases or health statuses and unveil the most representative species between different groups, we proceeded with a genus and species-level indicator species analysis (**Figure 3**). At the genus level, indicator genera identified for the Normal group included *Bifidobacterium*, *Faecalibacterium*, *Roseburia*, *Lachnospira*, *Coprococcus*, *Methyloversatilis*, *Hydrogenophaga*, and *Ruminococcus gnavus group* (**Figure 3A**). Indicator genera for the HSIL group comprised *Allobaculum*, *Aerococcus*, *Ochrobactrum*, *Megasphaera*, *Coriobacteriaceae_UCG-002*, *Fastidiosipila*, and *Castellaniella* (**Figure 3A**). In contrast, indicator genera of the CC group were *Pseudomonas*, *Porphyromonas*, *Peptoniphilus*, *Campylobacter*, *Halomonas*, and *Fenollaria* (**Figure 3A**).

At the species level, we also identified indicator species for the Normal group such as *Bifidobacterium longum*, *Streptococcus anginosus*, and *Streptococcus salivarius*, for the HSIL group such as *Aerococcus christensenii*, *Clostridiales_bacterium*, *Dialister micraerophilus*, *Sneathia sanguinegens*, and *Prevotella colorans*, and for the CC group including *Campylobacter ureolyticus*, *Porphyromonas asaccharolytica*, *Prevotella bergensis*, *Peptoniphilus duerdenii*, *Peptoniphilus coxii*, *Dialister propionicifaciens*, *Anaerococcus lactolyticus*, *Bacillus* sp*orothermodurans*, and *Bacillus oleronius* (**Figure 3B**).

### 3.4 Potential functions of the bacterial community

**Figure 4:**
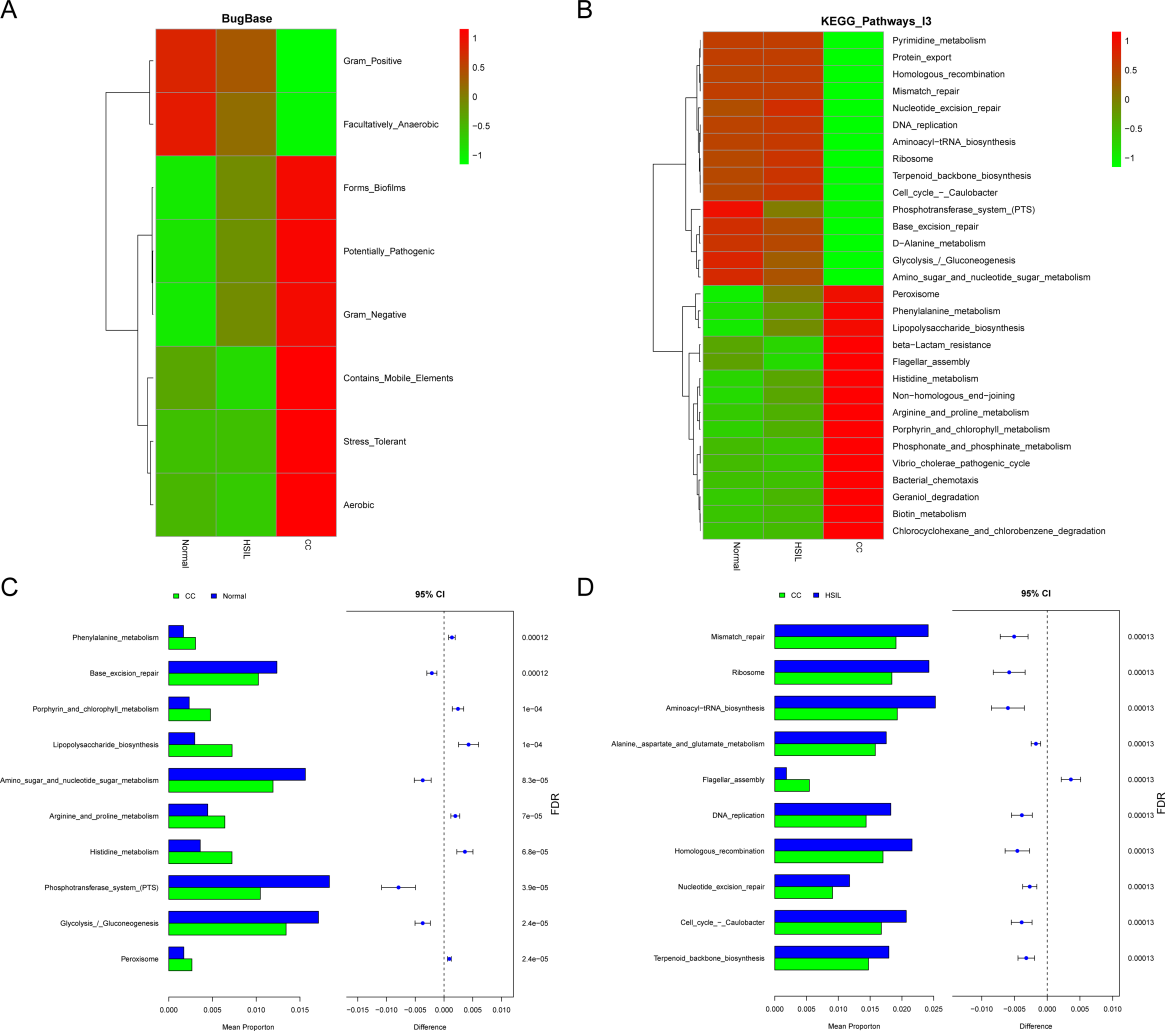
Functional prediction of the bacterial community. **(A)** Heatmap of bugbase predictions for the different groups, with colors corresponding to the z-score values of phenotype abundance. **(B)** Functional abundance of the top 30 KEGG pathways predicted based on picrust2 for different groups. **(C, D)** Top 10 differential KEGG pathways identified between CC vs Normal **(C)** and CC vs HSIL **(D)**, where FDR-corrected *P-value* < 0.05 indicate significant inter-group differences.

The results from microbial community phenotypes predicted based on BugBase revealed that that the abundance of Gram_Positive and Facultatively_Anaerobic species was higher in the Normal group, whereas the abundance of Aerobic, Stress_Tolerant, Contains_Mobile_Elements, Gram_Negative, Potentially_Pathogenic, and Forms_Biofilms species was higher in the CC group (**Figure 4A**). Further predictions of KEGG pathway abundances for each sample group using PICRUSt2 demonstrated notable differences. Fundamental biological functions such as amino sugar and nucleotide sugar metabolism, Aminoacyl-tRNA biosynthesis, and Phosphotransferase system (PTS) were more abundant in the Normal group, while pathways including beta-Lactam resistance, Flagellar assembly, Vibrio cholerae pathogenic cycle, Peroxisome, Phenylalanine metabolism, and Lipopolysaccharide biosynthesis were more prevalent in the CC group (**Figure 4B**). Notably, the pathway Vibrio cholerae pathogenic cycle indicated homology of virulence genes in the microbiota, since *V. cholerae* generally does not colonize or infect the cervix. Stamp analysis revealed that KEGG pathways in the CC group, such as Amino sugar and nucleotide sugar metabolism, Aminoacyl-tRNA biosynthesis, and Phosphotransferase system (PTS), were significantly lower compared to the Normal group. While pathways such as beta-Lactam resistance, Flagellar assembly, Vibrio cholerae pathogenic cycle, Penicillin and cephalosporin biosynthesis, and Peroxisome were significantly higher in the CC group than the Normal (**Figure 4C**; **Supplementary Table 3**) and HSIL group (**Figure 4D**; **Supplementary Table 4**). Furthermore, there were no significant differences in abundance of pathways between the Normal group and the HSIL group (**Supplementary Table 5**).

### 3.5 Identification of vaginal metabolomic signatures

**Figure 5:**
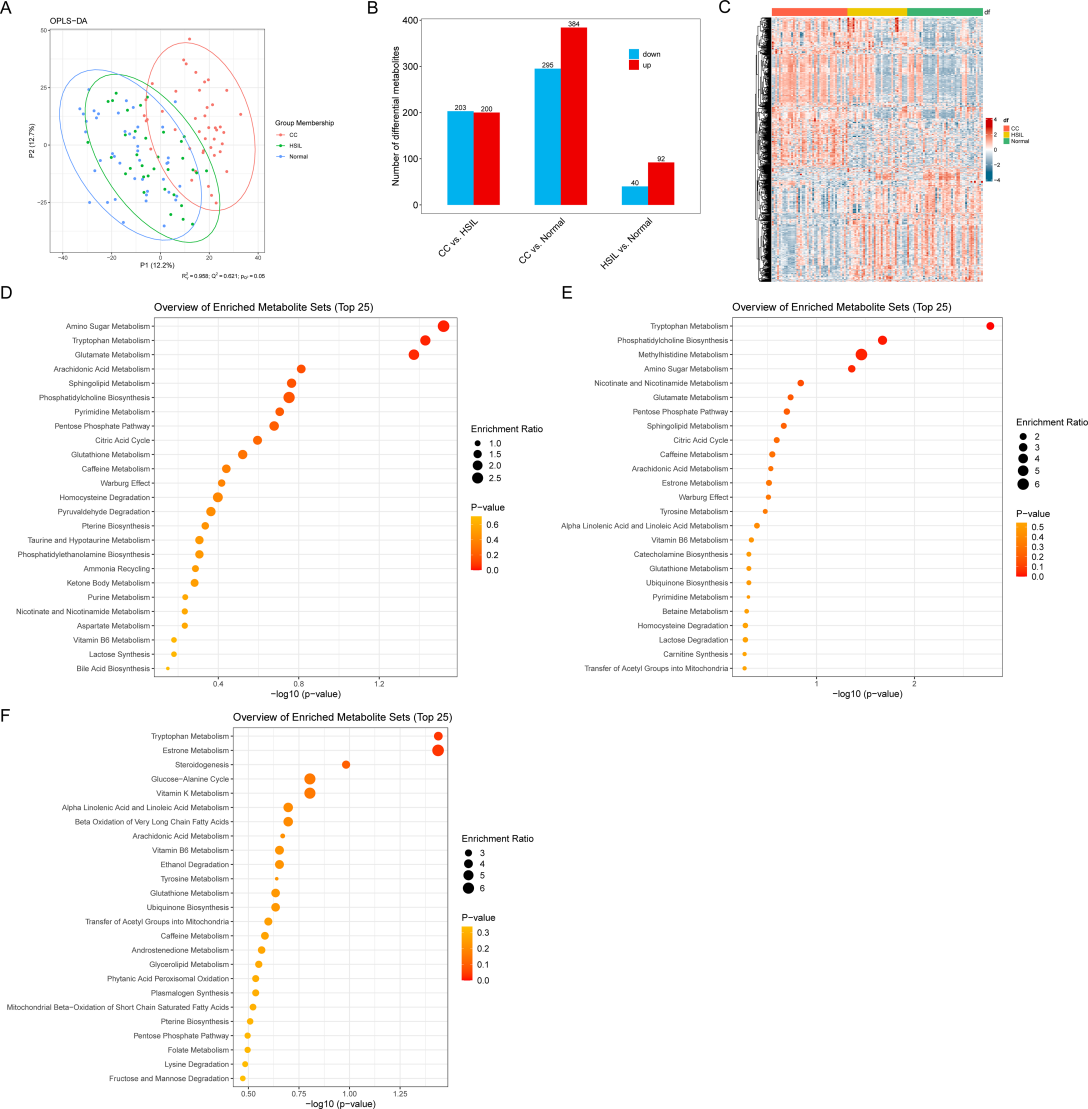
Vaginal metabolic alterations. **(A)** OPLS-DA score plot of different groups. **(B)** Number of differential metabolites for different comparisons. **(C)** Heatmap representing the abundance of differential metabolites. **(D–F)** Enrichment analysis results for differential metabolites in CC vs. HSIL, CC vs. Normal, and HSIL vs. Normal. Node size represents the number of differential metabolites in each pathway.

The score plot of the OPLS-DA model vividly illustrates significant separation between inter-group samples, indicating marked differences in cervical swab metabolomes across the various groups (**Figure 5A**). We identified a set of differential metabolites associated with cervical cancer, among which the number of differential metabolites between HSIL and Normal was relatively modest (51) (**Figure 5B**; **Supplementary Table 6**). In contrast, the number of differential metabolites in the CC vs. HSIL and CC vs. Normal comparison groups was larger, with a notably higher number of up-regulated metabolites compared to down-regulated metabolites (**Figures 5B, C**; **Supplementary Table 6**).

Enrichment analysis further revealed insights into the biological pathways associated with these differential metabolites. Specifically, the differential metabolites in CC vs. HSIL were enriched in pathways related to Amino Sugar Metabolism, Tryptophan Metabolism, and Glutamate Metabolism (**Figure 5D**). Meanwhile, the differential metabolites in CC vs. Normal were enriched in pathways associated with Tryptophan Metabolism, Phosphatidylcholine Biosynthesis, Methylhistidine Metabolism, and Amino Sugar Metabolism (**Figure 5E**). Lastly, the differential metabolites in HSIL vs. Normal exhibited enrichment in pathways such as Tryptophan Metabolism and Estrone Metabolism (**Figure 5F**).

### 3.6 Interaction of key microbiota and metabolites

**Figure 6:**
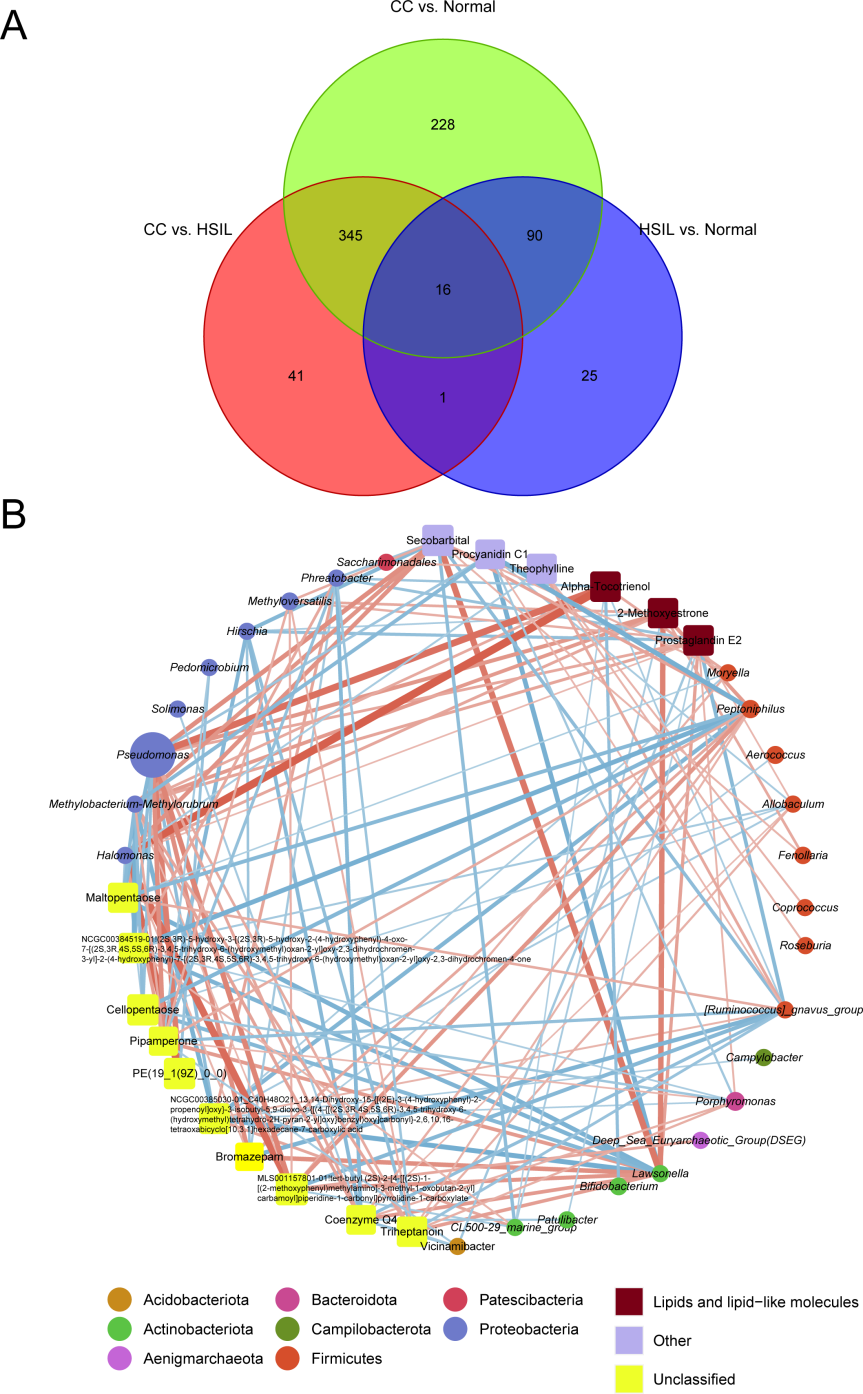
Visualization of the interaction network highlighting key microbiota-metabolite associations. **(A)** Venn diagram showing differential metabolites from three comparison groups (HSIL vs. Normal, CC vs. HSIL, and CC vs. Normal). **(B)** The interaction network of key microbiota and metabolites. Red and blue lines represent positive and negative correlations, respectively. Circles and squares represent microbiota and metabolites, respectively, with the size of the nodes proportional to the relative abundance of key microbiota or metabolites. Node colors correspond to taxonomic classifications at the phylum level for microbiota or metabolite categories.

By intersecting differential metabolites from three comparison groups (HSIL vs. Normal, CC vs. HSIL, and CC vs. Normal), a total of 16 key metabolites were identified. Spearman correlation coefficients between these key differential metabolites and indicative species were calculated to construct an interaction network between key microbial taxa and metabolites (**Figure 6A**).

Pseudomonas was among the microbial taxa with higher abundance in the interaction network, while metabolites such as Alpha-Tocotrienol, 2-Methoxyestrone, PGE2, Secobarbital, Procyanidin C1, and Theophylline exhibited higher abundance (**Figure 6B**). Notably, the top microbial feature in random forest model, *Porphyromonas*, exhibited positive correlations with key metabolites, including PGE2, Triheptanoin, and Coenzyme Q4, while displaying negative correlation with Cellopentaose (**Figure 6B**). Meanwhile, *Pseudomonas*, *Halomonas*, and *Methylobacterium-Methylorubrum* which belongs to *Proteobacteria* phylum displayed significant positive correlations with lipid metabolites such as Alpha-Tocotrienol, 2-Methoxyestrone, and PGE2, while *Lawsonella* showed apparent co-occurrence with Secobarbital (**Figure 6**). Conversely, *[Ruminococcus]_gnavus_group* demonstrated negative correlations with a plethora of metabolites (**Figure 6B**).

### 3.7 Predictive model based on vaginal microbial and metabolomic signatures

**Figure 7:**
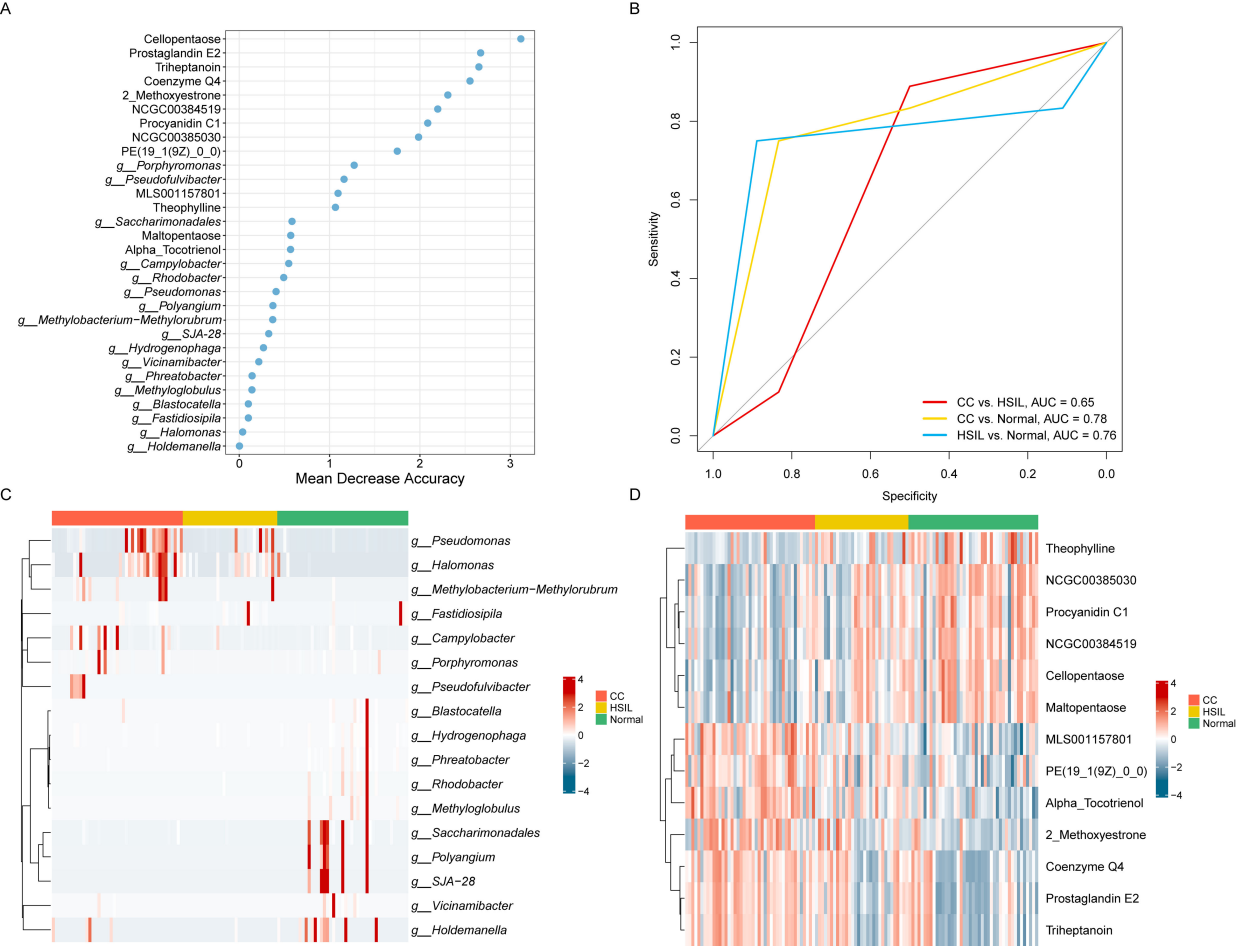
Prediction model for cervical cancer lesions. **(A)** Top 30 key metabolites/microbes as the optimal feature set of a random forest model based on ten-fold cross-validation. **(B)** ROC curve of the random forest model on the test dataset. **(C)** Heatmap representing the relative abundance of key microbes. **(D)** Heatmap representing the relative abundance of key metabolites.

To assess the predictive value of taxonomical features of cervical disease-associated microbiota and key metabolites, we employed a Random Forest Model (RFM) to discriminate among CC, HSIL, and Normal groups (**Figure 7**). Based on ten-fold cross-validation accuracy, we identified the top 30 microbiota/metabolites as the optimal feature set, among which key known metabolites, including Cellopentaose, Prostaglandin E2 (PGE2), Triheptanoin, Coenzyme Q4, 2-Methoxyestrone, Procyanidin C1, and Theophylline, along with genera *Porphyromonas* and *Pseudofulvibacter*, ranked highest in the model (**Figure 7A**).

Leveraging these 30 features, relatively high accuracy on the training set, with an average AUC of 70.00% (95% CI, 60.49%-79.51%) for CC vs. HSIL, 61.90% (95% CI, 53.04%-70.77%) for HSIL vs. Normal, and 74.17% (95% CI, 64.54%-83.79%) for CC vs. Normal, demonstrating high sensitivity and specificity in discriminating cervical cancer (CC) from Normal controls were achieved. Importantly, robust performance was observed on the test set, with AUC values of 64.81%, 75.93%, and 78.47% for CC vs. HSIL, HSIL vs. Normal, and CC vs. Normal, respectively (**Figure 7B**). Notably, the top key microbial features including *Porphyromonas* and *Pseudofulvibacter* showed a progressively increasing trend from normal to HSIL to CC (**Figure 7C**). The top key metabolites features including PGE2, Triheptanoin, and Coenzyme Q4 also showed a progressively increasing trend from normal to HSIL to CC, while Procyanidin C1, and Theophylline showed a progressively decreasing trend (**Figure 7D**). A simplified model using Porphyromonas, Pseudofulvibacter, PGE2, Triheptanoin, and Coenzyme Q4 achieved an AUC of 0.74 (**Supplementary Figure 2**), demonstrating the clinical feasibility of targeted biomarker panels.

## 4 Discussion

In this study, 16S rRNA amplicon sequencing and metabolomics approaches were utilized to investigate microbial and metabolic changes across normal, HSIL, and CC groups, aiming to identify key biomarkers and associated pathogenic mechanisms during aggravation of cervical neoplasia.

At the microbial level, the predominant genera with high abundance were mainly *Firmicutes*, *Proteobacteria*, *Actinobacteriota*, and *Bacteroidota* (**Figure 2A**), all of which have been reported in relevant vaginal microbiota studies (Diop et al., 2019). In this study, we observed increased microbial diversity in the CC group (**Figures 1B, C**) and a decrease in *Lactobacillus* (**Figure 2E**), consistent with previous findings of highly diversified vaginal microbiota and reduced *Lactobacillus* (Mitra et al., 2015; Sharifian et al., 2023; Wu et al., 2023; Li et al., 2024), further supporting their association with cervical cancer. Specifically, the abundance of *L. iners* was lower in CC compared to the other two groups (**Figure 2C**). The abundance of *Pseudomonas* was found to be higher in CC group, which is in accordance with previous studies (Li et al., 2024). Previous studies have indicated that the metabolite lactic acid produced by *L. iners* can activate the Wnt signaling pathway via the lactate-Gpr81 complex, thereby increasing the core fucosylation level in epithelial cells and suppressing the proliferation and migration of CC cells (Fan et al., 2021). One of the pivotal factors associated with cervical cancer is microbial dysbiosis (Mulato-Briones et al., 2024). Consistently, functional prediction in this study indicated an elevated abundance of Potentially_Pathogenic and Stress_Tolerant microbiota in the CC group compared to Normal group (**Figure 4**).

Metabolite enrichment analysis showed that the differential metabolites in all comparison groups were significantly associated with tryptophan metabolism (**Figures 5D–F**). Previous studies have indicated that microbial tryptophan metabolism modulates host immunity, impacting the progression of intestinal and extraintestinal disorders, such as inflammatory bowel disease (Liu et al., 2022). Additionally, tryptophan metabolism promotes the growth and invasion of gliomas while suppressing anti-tumor immune responses (Platten et al., 2012; Xu et al., 2021).

A random forest model was implemented to identify key microbial and metabolite biomarkers. Among these, the top two microbial genera included *Porphyromonas* and *Pseudofulvibacter*, both belonging to the phylum *Bacteroidota* (**Figure 7A**). Importantly, the *Bacteroidota* has been established as a characteristic feature within the context of CC (Fan et al., 2021). Furthermore, the *Porphyromonas* genus has been identified as important cervical cancer (CC) biomarkers in previous studies (Wu et al., 2023). It is noteworthy that the abundance of *Porphyromonas* and *Pseudofulvibacter* gradually increased from normal to HSIL and CC stages (**Figure 7C**). Karen V. Lithgow et al. demonstrated that vaginal *Porphyromonas* disrupt coagulation and extracellular matrix in the cervicovaginal niche (Lithgow et al., 2022). Studies have shown that *P. gingivalis* promotes local tissue destruction by inducing inflammatory processes (Katz et al., 2002; Takeuchi et al., 2019). Therefore, this species may contribute to a pro-inflammatory environment conducive to cervical cancer development, warranting further investigation into its role in CC pathogenesis.

Furthermore, metabolites identified as important features in the random forest model included Cellopentaose, PGE2, Triheptanoin, and Coenzyme Q4 (**Figure 7A**). Among these, PGE2 serves as a pivotal inflammatory mediator, exerting crucial roles in inflammatory responses (Kalinski, 2012; Serhan and Levy, 2018). Simultaneously, PGE2 acts on both autocrine and paracrine mechanisms to enhance tumor cell proliferation and survival, promote angiogenesis, and induce metastasis during cancer progression (Finetti et al., 2020). Besides, PGE2 level was significantly positive correlated with the abundance of *Porphyromonas* genus (**Figure 6B**). It is plausible to hypothesize that key microorganisms such as *Porphyromonas* within the vaginal environment induce inflammatory processes, thereby facilitating the occurrence and progression of cervical neoplasia.

The random forest model, as a highly effective and commonly used machine learning model, is widely applied in multi-omics research due to its ability to handle high-dimensional data, complex interaction effects, and resistance to overfitting (Diaz-Uriarte and Alvarez de Andres, 2006). However, integrating metabolomic data and microbial abundance and metabolomic data solely might overlook factors associated with the host. Due to incomplete patient medical records, there is a lack of data on smoking history, immune status and environmental exposures, as well as a small sample size in each subgroup after stratification, which has not been adequately considered in the current study regarding their impact on changes in the vaginal microbiome. It would be beneficial to further expand the sample size and fully collect and consider various clinical factors, such as age stratification, disease staging, menopausal status, smoking history, immune status and environmental exposures, *etc*. Additionally, further exploration of more complex model selection, such as incorporating latent variable models or multi-view models (Oshrit et al., 2024), will help to more comprehensively account for the complex relationships between the microbiome, metabolome, and host factors, thereby enhancing the predictive power and interpretability of the predictive model.

Classifying the microbiota into CSTs can provide additional insights into the overall microbial landscape and its association with disease states. The primary subCSTs identified in the present study cohort were III-A, III-B, IV-B, and IV-C0 (**Figure 2D**). Prior studies have also reported on datasets from four distinct countries/regions, revealing varying dominant subCSTs within their respective cohorts (Wu et al., 2023). This observation underscores the high variability in the microbiome composition and the necessity for large-scale studies to comprehensively elucidate these variations. In our future expanded cohort study, we will incorporate a CSTs analysis into our samples and discuss the implications of CST distributions in relation to disease progression. We believe this will help to contextualize our findings within the broader framework of vaginal microbiome research and provide a more comprehensive understanding of microbial dynamics.

There are several limitations in this study. Firstly, factors such as prior human papillomavirus (HPV) infection and HPV genotyping were not taken into account. This is because all cases in the HSIL and CC groups were infected with HPV, with most of them demonstrating single or multiple high-risk HPV infections. Persistent high-risk HPV infection has been shown to be closely associated with squamous intraepithelial lesions and cervical cancer (Munoz et al., 2003; Liu et al., 2020; So et al., 2020). Besides, complex interplay between HPV and the cervicovaginal microbiome in cervical carcinogenesis has been reported (Hong et al., 2023; Megan et al., 2024). Secondly, the low-grade SIL group was not included in the study cohort, which is crucial to elucidate the mechanisms during the progressive transition of cervical cancer. However, our preliminary research indicated that low-grade SIL shares a microbiota composition similar with that of healthy individuals (Zhai et al., 2021). Furthermore, experimental validation of *Porphyromonas* and PGE2 as an important biomarker and the role of *Porphyromonas* in inducing inflammatory responses was lacking. In future research, incorporating HPV-related factors and the LSIL group, along with corresponding experimental validation, may contribute to a better understanding of the role of the bacterial microenvironment in HPV persistence and cancer progression. Targeting specific vaginal microbiota associated with the pathogenesis could improve women’s health status and aid in the early detection and prevention of cervical precancerous lesions.

## Data Availability

The datasets presented in this study can be found in online repositories. The names of the repository/repositories and accession number(s) can be found in the article/**Supplementary Material**.

## Supplementary material

Supplementary Figure 1 Age distribution violin plots of Normal, HSIL, and CC groups.

Supplementary Figure 2 ROC curves of simplified biomarker including *Porphyromonas*, *Pseudofulvibacter*, PGE2, Triheptanoin, and Coenzyme Q4.
