# Supplementary figures and images for: Integrated analysis of microbiome and metabolome reveals insights into cervical neoplasia aggravation in a Chinese cohort
Source: Front Cell Infect Microbiol. 2025 May 8;15:1556153. doi: 10.3389/fcimb.2025.1556153 (PMC12095210; doi:10.3389/fcimb.2025.1556153)

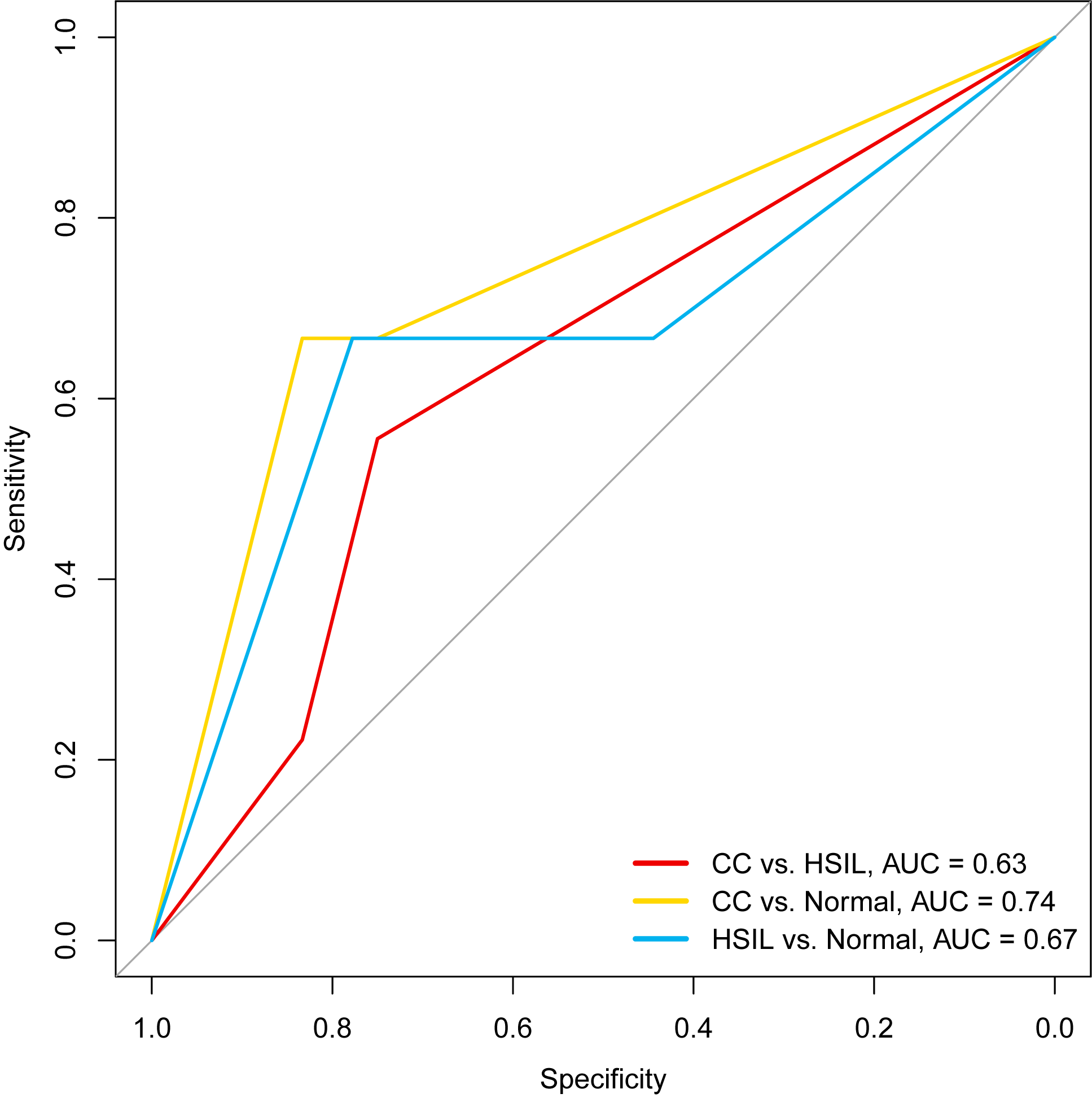

Supplement: Supplementary Figure 1 — Age distribution violin plots of Normal, HSIL, and CC groups. [file Image1.tif]

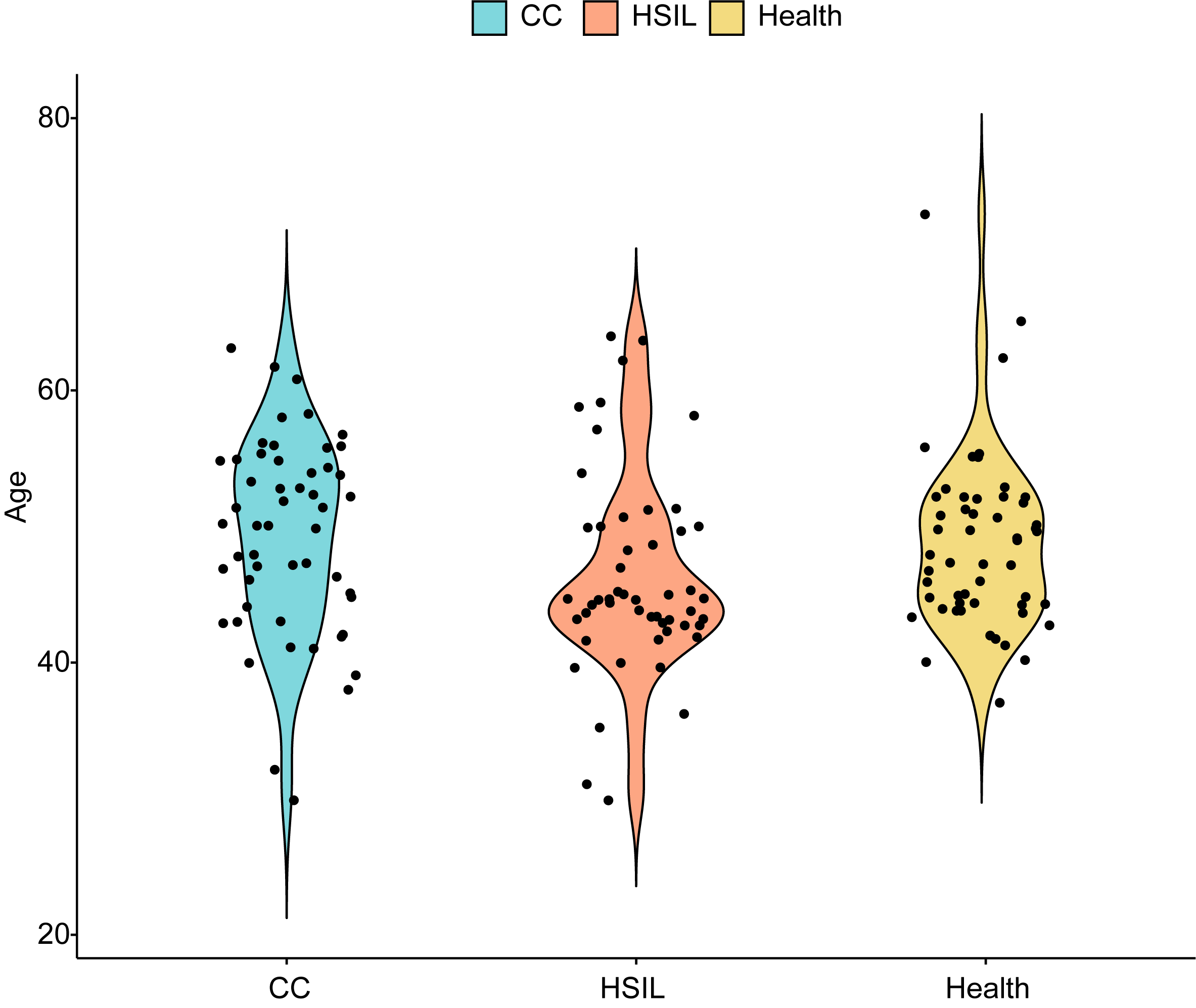

Supplement: Supplementary Figure 2 — ROC curves of simplified biomarker including Porphyromonas, Pseudofulvibacter, PGE2, Triheptanoin, and Coenzyme Q4. [file Image2.tif]
